# Complete Genome Sequence and Annotation of Corynebacterium singulare DSM 44357, Isolated from a Human Semen Specimen

**DOI:** 10.1128/genomeA.00183-15

**Published:** 2015-03-26

**Authors:** Madlen Merten, Karina Brinkrolf, Andreas Albersmeier, Yvonne Kutter, Christian Rückert, Andreas Tauch

**Affiliations:** Institut für Genomforschung und Systembiologie, Centrum für Biotechnologie (CeBiTec), Universität Bielefeld, Bielefeld, Germany

## Abstract

Corynebacterium singulare DSM 44357 is a urease-positive microorganism isolated from human semen. The complete genome sequence of C. singulare DSM 44357 comprises 2,830,519 bp with a mean G+C content of 60.12% and 2,581 protein-coding genes. The deduced antibiotic resistance pattern of this strain includes macrolides, lincosamides, aminoglycosides, chloramphenicol, and tetracyline.

## GENOME ANNOUNCEMENT

The human skin is a complex ecosystem colonized by a broad collection of microorganisms that are adapted to the specific niches they inhabit (1, 2). Systematic metagenomic analyses of the human skin microbiota have confirmed that corynebacteria compose a significant part of the normal microflora, particularly in moist areas of the human skin (3, 4). A recent search in the GenBank sequence database (5) with the BLASTn tool (6) and the 16S rRNA gene of Corynebacterium singulare DSM 44357 (7) as a query sequence revealed 99% identity to more than 170 operational taxonomic units (OTUs) from metagenomic studies of the human skin microflora (3, 4, 8). At least some of these OTUs may represent resident C. singulare strains, although this species is difficult to distinguish from Corynebacterium minutissimum when only comparing their 16S rRNA gene sequences (7, 9). Moreover, two C. singulare strains were recovered from a blood specimen and a semen specimen from two patients (7), and one C. singulare isolate was detected in a biofilm present on the outer spray plate surface of a domestic showerhead (10). To gain insights into the gene repertoire of C. singulare, we sequenced the genome of the type strain DSM 44357 (7).

C. singulare DSM 44357 (IBS B52218, CCUG 37330; CIP 105491) was obtained from the Leibniz Institute DSMZ (Braunschweig) and was grown in brain heart infusion broth-yeast extract medium at 37°C (11). Genomic DNA was purified with the Genomic-tip 500/G system and the Genomic DNA buffer set (Qiagen). The Nextera DNA sample preparation kit (Illumina) was used to generate a DNA library that was sequenced in a 2 × 300 nucleotide paired-end run using the MiSeq reagent kit version 3 (600 cycles) and the MiSeq desktop sequencer (Illumina). Whole-genome shotgun sequencing of the C. singulare DNA resulted in 2,417,003 paired reads and 584,352,026 detected bases. The assembly of the paired reads was performed with the Roche GS *de novo* Assembler software (release 2.8) and yielded 27 contigs in 12 scaffolds. The scaffolds were ordered by synteny analysis with the r2cat tool (12). The remaining gaps in the genome sequence were closed by PCR assays with the Phusion high-fidelity DNA polymerase (New England BioLabs) and by sequencing of the PCR products on an ABI 3730xl DNA analyzer (Applied Biosystems). The gap closure and finishing steps of this genome project were supported by the Consed finishing package (version 26) (13).

The chromosome of C. singulare DSM 44357 has a size of 2,830,519 bp with a mean G+C content of 60.12%. Gene finding was performed with Prodigal (14) and the functional annotation of the detected genes was supported by IMG/ER (15), revealing 2,581 protein-coding genes, 53 tRNA genes, and four rRNA operons. Antibiotic resistances of C. singulare DSM 44357 are presumably determinated by the macrolide and lincosamide resistance gene *erm*(X) (16, 17), the chloramphenicol exporter gene *cmx* (17, 18), the aminoglycoside resistance genes *aphA1-IAB* and *strA*-*strB* (19, 20), and the *texA-texB* tandem genes (formerly named *tetA-tetB*), encoding a heterodimeric corynebacterial tetracycline exporter (17, 21).

### Nucleotide sequence accession number.

This genome project has been deposited in the GenBank database under the accession no. CP010827.
